# Human Cytomegalovirus Strategies to Maintain and Promote mRNA Translation

**DOI:** 10.3390/v8040097

**Published:** 2016-04-13

**Authors:** Heather A. Vincent, Benjamin Ziehr, Nathaniel J. Moorman

**Affiliations:** Department of Microbiology & Immunology, Lineberger Comprehensive Cancer Center, University of North Carolina at Chapel Hill, Chapel Hill, NC 27599, USA; havincen@email.unc.edu (H.A.V.); bziehr@email.unc.edu (B.Z.)

**Keywords:** human cytomegalovirus, HCMV, protein synthesis, mRNA translation, translation initiation, eIF4F complex, mTOR signaling, eIF2α

## Abstract

mRNA translation requires the ordered assembly of translation initiation factors and ribosomal subunits on a transcript. Host signaling pathways regulate each step in this process to match levels of protein synthesis to environmental cues. In response to infection, cells activate multiple defenses that limit viral protein synthesis, which viruses must counteract to successfully replicate. Human cytomegalovirus (HCMV) inhibits host defenses that limit viral protein expression and manipulates host signaling pathways to promote the expression of both host and viral proteins necessary for virus replication. Here we review key regulatory steps in mRNA translation, and the strategies used by HCMV to maintain protein synthesis in infected cells.

## 1. Introduction

Viruses are completely reliant on the host translation machinery for the synthesis of viral proteins, since no virus encodes a ribosome. As a result, viral and host mRNAs must compete for access to ribosomes. Viruses must also counteract host defenses that inactivate the translation machinery after sensing viral infection. Human cytomegalovirus (HCMV) effectively antagonizes host defenses that limit viral protein expression. In addition, HCMV manipulates multiple host signaling pathways to ensure the continued synthesis of both host and viral proteins needed for virus replication. Thus, the interface between viral mRNAs and the host translation machinery serves as a critical determinant for successful HCMV infection. The purpose of this review is to summarize key steps in the regulation of mRNA translation, and strategies by which HCMV manipulates the host translation machinery to benefit virus replication.

## 2. The Scanning Model of Translation Initiation

Translation of mRNAs occurs in three steps: initiation, elongation and termination [1,2]. Translation initiation begins with the assembly of the eukaryotic initiation factor 4F (eIF4F) translation initiation complex on the 7-methyl guanosine cap (m^7^G cap) at the 5’ end of the mRNA. ([3,4,5] reviewed in [2]). The bound eIF4F complex recruits the 43S preinitiation complex (PIC), consisting of the 40S ribosomal subunit, the ternary complex and multiple initiation factors, to form the 48S initiation complex. The 48S complex then scans the 5’ untranslated region (UTR) of the mRNA until reaching the translation start codon, whereupon multiple initiation factors are released and the 60S ribosomal subunit is recruited [6]. Joining of the 40S and 60S ribosomal subunits to form the 80S ribosome marks the end of the initiation phase, and the beginning of elongation. Elongation is regulated by the eukaryotic elongation factor 1 (eEF1), which promotes binding of aminoacyl-tRNAs to the A-site of the ribosome, and eukaryotic elongation factor 2 (eEF2), which facilitates the translocation of peptidyl-tRNA from the A-site to the P-site of the ribosome [7,8,9]. Peptide elongation continues until a translation termination codon is encountered, whereupon translation ceases, the nascent peptide chain is displaced from the ribosome, and the ribosome disassembles [10,11]. The first step of translation, initiation, is the most regulated step ([12,13,14] reviewed in [15]) and is described in more detail below.

Formation of the eIF4F complex, which consists of eukaryotic initiation factors 4E (eIF4E), 4G (eIF4G) and 4A (eIF4A), bound to the 5’ m^7^G cap of an mRNA mediates the initiation of translation [2,16]. eIF4F assembly begins with binding of eIF4E to the m^7^G cap [3,17]. After binding the m^7^G cap, eIF4E recruits eIF4G, which acts as a scaffold protein that mediates the recruitment of the eIF4A RNA helicase, completing the assembly of the eIF4F complex [18,19]. eIF4G also coordinates the interaction of the eIF4F complex with additional factors bound to the mRNA, such as the poly(A) binding protein (PABP), that enhance translation initiation [2,16,18,19,20].

Prior to binding the eIF4F complex, multiple initiation factors associate with the 40S ribosomal subunit to prepare for translation initiation. Together, the 40S subunit and its associated factors are referred to as the 43S PIC ([21,22] reviewed in [23]). The 43S PIC contains the 40S ribosomal subunit, multiple initiation factors (e.g., eIF1, eIF1A, eIF3, and eIF5), and a ternary complex composed of the eukaryotic initiation factor 2 (eIF2), guanosine triphosphate (GTP) and a charged methionyl-tRNA (tRNA^Met^) [24]. eIF3 plays a critical role during 43S assembly, acting as a scaffold to recruit multiple initiation factors to the 40S ribosomal subunit [25]. These factors affect ribosome scanning, eIF4A helicase activity, and fidelity of start codon recognition once associated with the eIF4F complex on the 5’UTR of the mRNA [25,26].

Recruitment of the 43S PIC to the mRNA is mediated via an interaction between the eIF4G subunit of the eIF4F complex and eIF3 within the 43S PIC [27]. Together, the 43S PIC, the eIF4F complex, and the bound mRNA constitute the 48S initiation complex. Once assembled, the 48S complex scans the 5’UTR of the mRNA until reaching the translation “start” codon [28]. The eIF4A helicase, whose activity is stimulated by binding to eIF4B [29], unwinds RNA structures that would otherwise impede 48S scanning [28,30]. Additional RNA helicases such as DDX3, DHX9 and DHX29 also facilitate scanning through areas with high RNA secondary structure [31,32,33,34]. Scanning ceases upon recognition of a translation start site, which almost always consists of an AUG methionine codon in a favorable sequence context (*i.e.*, Kozak sequence [35]). The combined actions of the eIF1, eIF1A and eIF5 initiation factors position the tRNA^Met^ over the AUG codon, triggering hydrolysis of eIF2-bound GTP and release of a subset of initiation factors [6]. eIF5 directs subsequent joining of the 40S and 60S ribosomal subunits to form a functional 80S ribosome, after which peptide elongation commences [36]. Elongation continues until a translation termination or “stop” codon is encountered, at which point eukaryotic release factor 1 (eRF-1) together with eukaryotic release factor 3 (eRF-3) terminates elongation by displacing the nascent peptide from the ribosome [10,11].

## 3. Alternative Translation Initiation Mechanisms

Translation of some viral mRNAs does not require 40S ribosomal subunit scanning. Instead, ribosomes are recruited to the site of translation initiation, often with limited or no ribosome scanning, by specific RNA sequences/structures called internal ribosome entry sites (IRESs) ([37,38] reviewed in [39]). Ribosome recruitment to IRESs often requires only a subset of translation initiation factors. For example, translation initiation from the poliovirus IRES is independent of the eIF4E cap binding protein, but requires eIF4A, eIF4G and the 43S PIC [40]. On the other end of the spectrum, the cricket paralysis virus (CrPV) IRES requires only 40S and 60S ribosomal subunits to initiate translation [41,42]. Although these and other IRESs require differing factors to initiate translation, RNA secondary and tertiary structure is indispensable for the recruitment of ribosomal subunits to IRES elements [43].

In addition to initiation factors, IRES activity can be enhanced through binding of IRES trans-activating factors, or ITAFs [44]. Two cellular factors consistently function as ITAFs, the polypyrimidine tract binding protein (PTB) and the lupus autoantigen (La). PTB is an RNA binding protein that directly interacts with RNA secondary structures to promote RNA folding and maintain IRES structures [45,46]. The La protein binds the 5’UTR of multiple viruses, including poliovirus, human immunodeficiency virus (HIV), hepatitis C virus (HCV), and influenza virus [47]. Binding of La to IRES sequences stimulates recruitment of the 40S ribosomal subunit, possibly to promote correct start codon usage [48]. Both proteins are critical for the activity of multiple viral IRESs ([49,50] reviewed in [51]) and promote IRES driven translation during HCV, poliovirus and rhinovirus infection [52,53]. 

## 4. Signaling Pathways Regulating Translation Initiation

### 4.1. mTOR Signaling 

**Figure 1:**
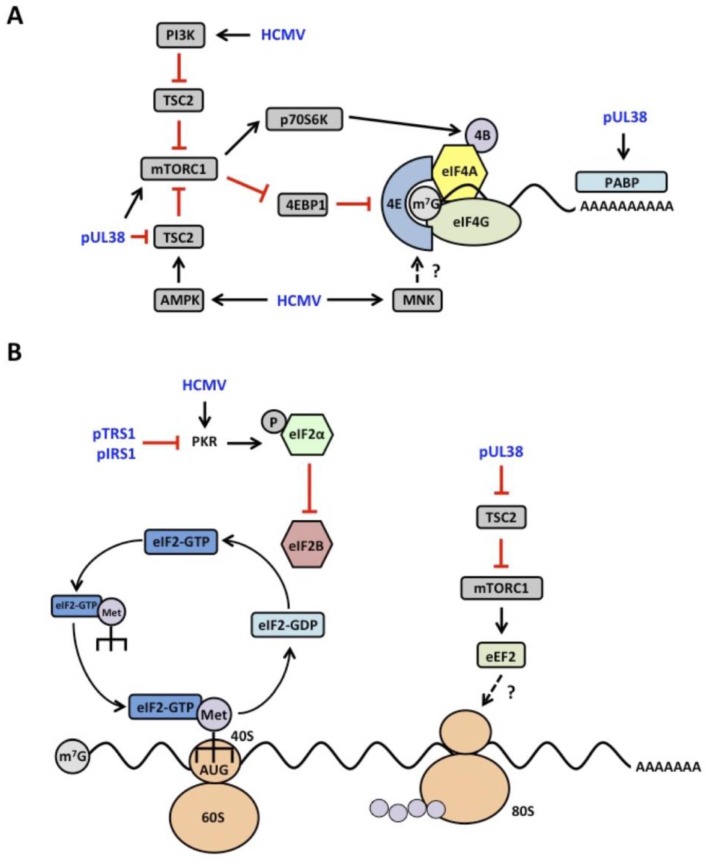
**Schematic of human cytomegalovirus (HCMV) manipulation of translation initiation and elongation.** (**A**) HCMV stimulates eIF4F formation and activity through multiple mechanisms. Infection increases levels of eIF4F components (eIF4E, eIF4G, eIF4A) and poly(A) binding protein (PABP). HCMV also promotes eIF4F assembly by activating mammalian target of rapamycin complex 1 (mTORC1), which phosphorylates and inhibits the eIF4F antagonist 4EBP1. The HCMV UL38 protein prevents inactivation of mTORC1 by inhibiting the tuberous sclerosis complex (TSC). pUL38 also stimulates mTORC1 activity through a TSC2-independent mechanism. mTORC1 activates p70S6K, which phosphorylates eIF4B to increase eIF4A helicase activity. HCMV infection activates the PI3K pathway to promote mTORC1 activation, and may also regulate translation through activation of MNK kinases (**B**) Translation initiation and elongation are maintained during HCMV infection. Inhibition of eIF2α phosphorylation ensures the regeneration of ternary complexes and continued rounds of translation initiation. Levels of eEF2 increase during infection through a UL38-dependent mechanism and may promote translation during HCMV infection.

The target of rapamycin (TOR) kinase is conserved throughout eukaryotes, where it plays a critical role in modulating protein synthesis in response to the intracellular and extracellular environment ([54,55] reviewed in [1]). In mammalian cells, mTOR (mammalian TOR) is found in two complexes: the mTORC1 and mTORC2 complexes [56,57]. While the two complexes share several subunits, each complex has unique defining components. mTORC1 contains the protein regulatory-associated protein of mTOR (RAPTOR), while the presence of rapamycin-insensitive companion of mTOR (RICTOR) defines the mTORC2 complex [58,59]. mTORC1 plays a central role in regulating translation initiation by controlling the assembly of the eIF4F complex [60]. In the hypo-phosphorylated state, the eIF4E-binding protein 1 (4EBP1) binds eIF4E and prevents binding to eIF4G, thereby limiting eIF4F formation [61]. Phosphorylation of 4EBP1 by mTORC1 reduces the affinity of 4EBP1 for eIF4E, allowing efficient eIF4F complex formation and increasing overall levels of protein synthesis (Figure 1A) [62].

mTORC1 also promotes protein synthesis by phosphorylating and activating the 70 kDa ribosomal protein S6 kinase (p70S6K). Active p70S6K phosphorylates several factors involved in translation, such as eIF4B and eukaryotic elongation factor 2 kinase (eEF2K) [63,64]. When phosphorylated, eIF4B binds eIF4A and increases its helicase activity, resulting in more efficient 48S scanning through 5’UTRs with significant secondary structure [65]. Like mTORC1 itself, p70S6K also phosphorylates and deactivates a translation repressor, eEFK2 [64,66]. eEFK2 phosphorylates and inactivates eukaryotic elongation factor 2 (eEF2), which stimulates the incorporation of amino acids into the growing peptide chain [67,68]. eEF2K phosphorylation of eEF2 prevents its association with the ribosome, thereby slowing the rate of elongation [7]. Thus, protein synthesis is induced through multiple mechanisms upon mTORC1 activation.

Protein synthesis is one of the most energy intensive cellular processes. Therefore mTORC1 is unsurprisingly subject to both positive and negative regulation in response to environmental cues. This regulation converges on the tuberous sclerosis complex (TSC), which negatively regulates mTORC1 activity [69,70]. The heterodimeric TSC consists of the tuberous sclerosis 1 (TSC1) and tuberous sclerosis 2 (TSC2) proteins that together act as a GTPase activating protein (GAP) for the mTORC1 cofactor Rheb [71]. mTORC1 is activated by association with Rheb-GTP and repressed by Rheb-GDP. Increased TSC GAP activity stimulates hydrolysis of GTP bound to Rheb, and therefore inhibits mTORC1 activity [72]. Decreased nutrient availability leads to elevated AMP to ATP ratios that activate the AMP-regulated protein kinase (AMPK) [73]. Active AMPK phosphorylates TSC2, stimulating TSC GAP activity and inhibiting mTORC1 activity [74]. Conversely, when nutrients are plentiful, growth factor receptor signaling activates the phosphoinositide 3-kinase (PI3K), which phosphorylates and inhibits the TSC complex, thereby promoting mTORC1 activity and eIF4F assembly [75]. Thus the TSC complex integrates upstream signaling pathways to modulate mTORC1 activity and protein synthesis to match the nutrient availability within the cellular environment.

### 4.2. eIF2α Kinase Activation 

Another focal point in the regulation of translation initiation is the phosphorylation of the alpha subunit of eukaryotic initiation factor 2 (eIF2α). eIF2α is part of the trimeric eIF2 complex, which together with GTP and tRNA^Met^, forms the ternary complex associated with the 43S PIC [76]. Recognition of the AUG initiation codon by tRNA^Met^ stimulates the hydrolysis of eIF2-associated GTP. The resulting release of the free phosphate triggers eIF2-GDP release from the ribosome [77]. Eukaryotic initiation factor 2B (eIF2B) exchanges GDP for GTP in the eIF2 complex, allowing eIF2-GTP to form a new ternary complex and participate in subsequent rounds of initiation [78]. Phosphorylation of eIF2α by one of four eIF2α kinases (see below) greatly increases the affinity of eIF2B for eIF2, preventing eIF2B release and GTP exchange [79,80]. As eIF2α is present at a much greater concentration than eIF2B, even small increases in eIF2α phosphorylation can sequester essentially all of the available eIF2B, preventing the formation of new ternary complexes and resulting in a dramatic decrease in protein synthesis [81].

Similar to the mTOR signaling pathway, eIF2α kinases regulate protein synthesis in response to the intracellular environment. Four eIF2α kinases have been identified, each of which are activated in response to a distinct cellular stress (reviewed in [82]). The heme-regulated inhibitor (HRI) kinase phosphorylates eIF2α in response to high levels of reactive oxygen species, linking the rate of protein synthesis to the respiratory capacity of the cell [83]. Accumulation of unfolded proteins within the endoplasmic reticulum (ER) activates the PKR-like endoplasmic reticulum kinase (PERK) [84], temporarily inhibiting protein synthesis to allow the cell to properly fold or degrade accumulated unfolded proteins. The eIF2α kinase general control nonderepressible 2 (GCN2) is activated by binding to uncharged tRNAs that accumulate during amino acid deprivation, directly linking amino acid availability to the rate of protein synthesis [85,86]. Especially relevant to viral infections, protein kinase R (PKR) is activated by binding to double stranded RNAs (dsRNAs) produced during infection [87,88,89,90], and potently inhibits viral protein synthesis, and thus virus replication [80].

## 5. HCMV Manipulation of Translation Initiation

Unlike many viruses, HCMV does not limit host translation. Instead, overall levels of protein synthesis are maintained in HCMV-infected cells [91]. As both host and viral mRNAs rely on the same pool of ribosomes for their translation, HCMV mRNAs must efficiently compete with host transcripts for access to the translation machinery to ensure synthesis of viral proteins. In addition, the virus must circumvent antiviral defenses that otherwise limit protein synthesis upon infection. HCMV infection activates both antiviral defenses and stress response pathways, yet viral protein synthesis and replication remain unaffected (see below). While the full spectrum of strategies employed by HCMV to regulate host responses is beyond the scope of this review, examples of the strategies HCMV uses to maintain viral protein synthesis by counteracting host defenses and stress responses are described below. 

### 5.1 HCMV Infection Increases eIF4F Abundance and Activity during Infection

As discussed above, the eIF4F complex plays a critical role in translation initiation by recruiting the 43S PIC to the 5’ end of the mRNA. Recruitment of the eIF4F complex, particularly binding of the eIF4E subunit to the mRNA m^7^G cap, is thought to be the rate-limiting step of translation initiation [92]. Overall levels of eIF4F directly correlate with the level of protein synthesis within the cell. The sustained eIF4F-dependent translation of host mRNAs during HCMV infection suggests that the virus manipulates cellular signaling pathways to maintain eIF4F activity (Figure 1A). Consistent with this idea, *Walsh et al.* found that HCMV infection increases the abundance of eIF4F subunits and promotes eIF4F complex formation [91]. In addition, elevated levels of PABP stimulate eIF4F formation during infection [93]. The increase in eIF4F levels in infected cells is important for virus replication, as disrupting or inhibiting the eIF4F complex early during infection profoundly limits viral replication [94,95,96]. Similarly, an eIF4A helicase inhibitor suppresses HCMV replication *in vitro* when added at the time of infection [95]. This in part reflects the necessity for eIF4F-dependent translation of host mRNAs during infection, as depletion of several host proteins that require eIF4F for their expression reduced the production of progeny virus [97].

In addition to increasing the abundance of eIF4F subunits, HCMV activates signaling pathways that stimulate eIF4F complex formation. mTORC1 activity is increased in HCMV infected cells, promoting eIF4F formation through phosphorylation and inactivation of the 4EBP family of translational repressors (Figure 1A) [91,94,98]. During infection, mTORC1 activity is refractile to cellular stresses such as AMPK activation that typically decrease its activity [99,100,101]. In fact HCMV paradoxically requires increased activation of both AMPK and mTOR during infection for efficient replication [102]. Thus HCMV uncouples mTORC1 activity from negative regulatory cues to promote virus replication. The dichotomy of continued mTORC1 activity despite AMPK activation can be explained, in part, by the finding that the HCMV UL38 protein (pUL38) binds and inhibits the host TSC2 protein, preventing inhibition of mTORC1 in response to nutrient deprivation and AMPK agonists [103]. pUL38 also stimulates mTORC1 activity in a TSC2-independent manner, although the mechanism remains unclear (Figure 1A) [104]. Thus HCMV pUL38 severs the connection between AMPK and mTOR signaling, allowing for eIF4F formation and maintained levels of protein synthesis. 

HCMV infection also stimulates additional signaling pathways that potentially enhance translation during infection. The PI3K signaling pathway is stimulated during infection and increases mTORC1 activity [105,106]. Chemical inhibitors of PI3K limit HCMV replication [105], suggesting that PI3K signaling could play a role in stimulating translation in infected cells. HCMV infection also activates the MNK kinases [91], which phosphorylate eIF4E (Figure 1A). Phosphorylation of eIF4E is suggested to increase the rate of translation through an unknown mechanism. Inhibitors of the MNK kinases reduce HCMV replication [91], suggesting that MNK-dependent eIF4E phosphorylation potentially regulates protein synthesis during infection. Infection also increases the abundance of the critical translation elongation factor eEF2 in a UL38-dependent manner (Figure 1B). While the role of the above signaling changes in HCMV translation has not been demonstrated, their association with the control of translation in other contexts suggests a potential role in translation regulation during HCMV infection.

As suggested by the multiple mechanisms HCMV employs to induce and maintain mTORC1 activity, decreased mTOR activity or expression inhibits virus replication [94,95,96]. Depletion of mTOR, RICTOR or RAPTOR decreases HCMV replication, as do ATP-competitive inhibitors of mTOR kinase activity [98,107]. When added at the time of infection, mTOR inhibitors limit viral DNA accumulation and thus the transcription of HCMV late genes. However, mTOR inhibitors also prevent metabolic remodeling induced by HCMV during infection, thus the effects of the inhibitors are likely pleiotropic [108]. Although mTOR inhibitors limit virus replication when added at the start of infection, such drugs have little effect on viral protein synthesis, the association of viral mRNAs with polysomes or virus replication when added later in infection [96,107].

### 5.2. IRES Activity during HCMV Infection

Many viruses use IRES elements to ensure translation of viral mRNAs under stress conditions that limit host protein synthesis. The only IRES-like element identified to date in the HCMV genome is located adjacent to the UL138 open reading frame (ORF). The UL138 ORF is the most 3’ of four ORFs encoded on a polycistronic mRNA, suggesting that cap-mediated translation initiation of UL138 would be exceedingly inefficient. The UL138 IRES-like element directs internal initiation on the polycistronic pUL138 transcript and allows for increased UL138 protein synthesis under conditions of cell stress [109]. While the role of the UL138 IRES during infection is unknown, it likely plays a role in regulating HCMV latency, as pUL138 acts as a molecular switch that regulates virus reactivation [110]. Whether or not HCMV encodes additional IRES-like elements that allow for non-canonical translation initiation events remains to be determined. 

HCMV infection also stimulates the translation of at least one IRES-containing cellular mRNA needed for virus replication. Cells induce a coordinated response to the accumulation of unfolded proteins in the ER, aptly named the unfolded protein response (UPR). While induction of the UPR generally suppresses protein synthesis, the translation of a subset of mRNAs involved in resolving cell stress is selectively increased. While HCMV infection induces the UPR, downstream signaling pathways are selectively modulated to support virus replication [111,112]. One example is the increased IRES-dependent translation of the ER chaperone BiP (binding immunoglobulin protein) during infection, which likely supports HCMV replication by increasing the protein folding capacity of the ER [113,114]. HCMV stimulates BiP mRNA translation in part by increasing the expression of the La protein, a known ITAF for the BiP IRES [115]. Given the increase in La abundance during infection and the wide range of cellular and viral mRNAs that La interacts with, HCMV infection may stimulate the translation of additional IRES-containing mRNAs.

### 5.3. HCMV Regulation of eIF2α Kinases

HCMV infection generates cellular stresses that are potent activators of eIF2α kinases. Of the eIF2α kinases, the role of the antiviral PKR during infection is the best characterized. PKR is activated upon binding to double stranded RNAs (dsRNAs) generated during the early stage of HCMV infection [116]. Yet PKR activation and eIF2α phosphorylation are not observed at this time, suggesting HCMV actively prevents PKR activation. Using a screen to identify HCMV genes that rescue growth of a vaccinia virus mutant lacking its PKR antagonist E3L, the laboratory of Dr. Adam Geballe identified the HCMV TRS1 and IRS1 proteins (pTRS1 and pIRS1, respectively) as potent PKR antagonists [117]. Expression of either pTRS1 or pIRS1 is necessary for HCMV infection, as infection in the absence of both proteins results in an almost complete block to virus replication [116]. The first two thirds of TRS1 and IRS1 are encoded by the terminal repeats flanking the unique short region of the HCMV genome, and thus the first two thirds of pTRS1 and pIRS1 are identical [118]. This common amino-terminal region contains an unusual RNA binding domain [119], which functions to competitively inhibit PKR by binding to dsRNA ligands [120]. Both pTRS1 and pIRS1 also contain a PKR binding domain in their divergent C-terminal region [121]. The RNA and PKR binding domains of pTRS1 are both necessary to rescue replication of a vaccinia virus lacking the E3L protein [119,120]. However, the physical interaction with PKR appears to be most important for PKR inhibition during HCMV infection, as mutation or deletion of the pTRS1 PKR binding domain results in PKR activation in the absence of pIRS1 [122,123]. When PKR is depleted or deleted prior to infection HCMV replication is restored in the absence of pTRS1 and pIRS1 [122,123], suggesting that a major function of pTRS1/pIRS1 is to counteract inhibition of viral protein synthesis by PKR (Figure 1B).

Despite pTRS1/pIRS1 inhibition of PKR, phosphorylated eIF2α accumulates in infected cells during the late stage of HCMV infection [111,124]. Mammalian cells express three additional eIF2α kinases, PERK, HRI and GCN2 whose activation could account for the observed increase in eIF2α phosphorylation. While the potential role of HRI and GCN2 in HCMV-induced eIF2α phosphorylation is unknown, PERK is not the relevant eIF2α kinase as phosphorylated eIF2α accumulates during infection of PERK-depleted cells [112]. In any case, both host and viral mRNAs are translated despite the observed increase in eIF2α phosphorylation late in infection. It is currently unclear if eIF2α phosphorylation no longer restricts translation initiation late in infection, or if the extent of eIF2α phosphorylation is insufficient to suppress protein synthesis. Perhaps the increased abundance and activity of the host PP1 and PP2A phosphatases during infection [125] in conjunction with viral inhibitors of eIF2α kinases allow for efficient translation in the presence of multiple eIF2α stresses. Regardless, continued protein synthesis in the face of significant cellular stress suggests that HCMV encodes viral proteins that limit eIF2α kinase activity, or actively limit eIF2α phosphorylation in response to virus-induced stress. 

## 6. Unresolved Questions and Future Directions

As described above, HCMV employs a variety of strategies to counteract host defenses and maximize the translation of viral mRNAs. However, several recent studies suggest that our understanding of HCMV manipulation of the host translation machinery is incomplete. One unresolved question concerns the role of the eIF4F complex in HCMV protein synthesis. Disrupting or inhibiting the eIF4F complex at the start of infection decreases HCMV replication [94,95,96], correlating with a decrease in the translation efficiency of several host mRNAs needed for progression through the viral lytic cycle [97]. Yet as infection progresses, viral replication becomes increasingly resistant to mTOR inhibitors, even though these inhibitors continue to efficiently disrupt eIF4F complex formation [107]. Despite significantly reduced eIF4F abundance, HCMV mRNAs efficiently associate with polysomes even though the translation of several host mRNAs is simultaneously decreased [95]. How are viral mRNAs efficiently translated under eIF4F-limiting conditions? Perhaps the relatively high abundance of HCMV transcripts with structurally simple 5’UTRs allows them to compete efficiently for ribosomes when eIF4F is limiting. Another possibility is that a host or viral factor recruits residual eIF4F specifically to HCMV mRNAs. This may in part explain the finding that HCMV mRNAs as a group are more efficiently translated than host mRNAs during the later stages of infection [126]. Alternatively, an unknown host or viral factor might functionally substitute for components of the eIF4F complex. This could be a host factor that similarly facilitates translation of cellular mRNAs under eIF4F-limiting conditions. In any case, these results suggest that translation initiation on HCMV mRNAs differs from that on eIF4F-dependent host mRNAs. Exploring the mechanisms underlying this difference will likely expand our understanding of both cellular and viral translation mechanisms.

Another emerging question in the regulation of HCMV translation stems from the recent discovery that the coding potential of the viral genome has been greatly underestimated. Ribosome profiling (next generation sequencing of ribosome-protected mRNA fragments) of HCMV infected cells combined with mass spectrometry revealed that HCMV encodes over 750 polypeptides [127], as compared to previous estimates of approximately 200 protein coding regions [128,129]. How does HCMV encode such a wide array of peptides, and what is their function? In some cases the use of a near-cognate initiation codon drives translation from previously unrecognized ORFs, although the majority of the novel coding regions initiate with an AUG start codon. Many of the novel peptides were less than 30 amino acids in length, and therefore may not function as proteins in the commonly understood sense. However, short peptides encoded upstream of longer coding regions on the same mRNA can serve regulatory roles to modulate mRNA translation in response to the cellular environment. Small upstream open reading frames, or uORFs, are translation competent ORFs consisting of fewer than 30 codons located 5’ of a protein coding region on the same mRNA ([130,131] reviewed in [132]). Under normal conditions ribosomes initiate translation at the first AUG codon they encounter in a favorable sequence context (e.g., Kozak sequence [35]), and therefore translate uORFs rather than the downstream protein coding region. However, during periods of stress that limit ternary complex availability, uORFs can counterintuitively stimulate translation of the downstream coding region [133] through a poorly defined mechanism. Therefore these short, translation competent HCMV ORFs could act as translational regulators for adjacent protein coding regions, providing the virus a means to ensure continued viral protein expression under conditions of cellular stress.

Many new coding regions were also found internal or antisense to previously annotated ORFs, raising the question of how these novel ORFs are translated. Is each peptide-coding region contained on its own transcript? Or might HCMV extensively utilize polycistronic mRNAs to expand its coding capacity? Based on the relative paucity of confirmed IRES elements in herpesvirus genomes, it seems unlikely that internal ribosome entry can explain the diversity of the viral translatome. A more likely explanation for the diversity of viral polypeptides is that the complexity of the HCMV transcriptome has also been underestimated. Both strands of the viral genome are extensively transcribed [134,135,136], potentially explaining the presence of “antisense” coding regions in some ORFs. HCMV mRNA splicing is also more extensive than once thought [134], and alternative transcription start site (TSS) usage in some cases extends or truncates known reading frames [136]. Such extensive transcriptome complexity suggests that each HCMV polypeptide may arise from cap-dependent translation of a monocistronic mRNA, rather than through IRES-dependent translation of polycistronic messages. A more thorough characterization of the HCMV transcriptome will therefore shed light on the mechanisms regulating the translation of the expansive HCMV proteome.

While we know much about the control of mRNA translation during HCMV infection, much remains to be learned. The growing list of high-resolution techniques available to study protein synthesis should allow for a more complete picture of regulatory events controlling both host and viral mRNA translation during HCMV infection. Given the growing appreciation of the importance of translation regulation in multiple disease states, this knowledge may lead to new targets for novel therapeutics that inhibit viral protein synthesis, and thereby decrease HCMV disease.

## Abbreviations

The following abbreviations are used in this manuscript:

HCMV	human cytomegalovirus
eIF4F	eukaryotic initiation factor 4F
m^7^G cap	7-methyl guanosine cap
43S PIC	43S preinitiation complex
UTR	untranslated region
eEF1	eukaryotic elongation factor 1
eEF2	eukaryotic elongation factor 2
eIF4E	eukaryotic initiation factor 4E
eIF4G	eukaryotic initiation factor 4G
eIF4A	eukaryotic initiation factor 4A
PABP	poly(A) binding protein
eIF1	eukaryotic initiation factor 1
eIF1A	eukaryotic initiation factor 1A
eIF3	eukaryotic initiation factor 3
eIF4B	eukaryotic initiation factor 4B
eIF5	eukaryotic initiation factor 5
eIF2	eukaryotic initiation factor 2
tRNA^Met^	methionyl-tRNA
eRF-1	eukaryotic release factor 1
eRF-3	eukaryotic release factor 3
IRES	Internal Ribosome Entry Sites
ITAF	IRES trans-activating factor
PTB	polypyrimidine tract binding protein
La	lupus autoantigen
HIV	human immunodeficiency virus
HCV	hepatitis C virus
mTOR	mammalian target of rapamycin kinase
4EBP1	eIF4E-binding protein 1
p70S6K	70 kDa ribosomal protein S6 kinase
eEF2K	eukaryotic elongation factor 2 kinase
TSC	tuberous sclerosis complex
TSC1	tuberous sclerosis complex 1
TSC2	tuberous sclerosis complex 2
GAP	GTPase activating protein
AMPK	AMP-regulated protein kinase
PI3K	phosphoinositide 3-kinase
AKT	protein kinase B
eIF2α	eukaryotic initiation factor 2 alpha subunit
eIF2B	eukaryotic initiation factor 2B
HRI	heme-regulated inhibitor
ER	endoplasmic reticulum
PERK	PKR-like endoplasmic reticulum kinase
GCN2	general control nonderepressible 2
PKR	protein kinase R
dsRNA	double stranded RNA
UPR	unfolded protein response
BiP	binding immunoglobulin protein
ORF	open reading frame
uORF	upstream open reading frame
TSS	transcription start site
